# Hyperbaric oxygen therapy efficacy on mandibular defect regeneration in rats with diabetes mellitus: an animal study

**DOI:** 10.1186/s12903-023-02801-w

**Published:** 2023-02-15

**Authors:** Rodina H. Eldisoky, Salwa A. Younes, Samia S. Omar, Hagar S. Gharib, Tarek A. Tamara

**Affiliations:** 1 Department of Oral Biology, Faculty of Dentistry, Alexandria University, Alexandria, Egypt; 2 Naval Hyperbaric Medical Institute, Military Medical Academy, Alexandria, Egypt

**Keywords:** Hyperbaric oxygen therapy, Bone regeneration, Mandibular defects, Type I diabetes mellitus

## Abstract

**Background:**

This study aimed to investigate the influence of hyperbaric oxygen therapy on mandibular critical-sized defect regeneration in rats with experimentally induced type I diabetes mellitus. Restoration of large osseous defects in an impaired osteogenic condition such as diabetes mellitus is a challenging task in clinical practice. Therefore, investigating adjunctive therapies to accelerate the regeneration of such defects is crucial.

**Materials and methods:**

Sixteen albino rats were divided into two groups (n = 8/group). To induce diabetes mellitus, a single streptozotocin dosage was injected. Critical-sized defects were created in the right posterior mandibles and filled with beta-tricalcium phosphate graft. The study group was subjected to 90-min sessions of hyperbaric oxygen at 2.4 ATA, for 5 consecutive days per week. Euthanasia was carried out after 3 weeks of therapy. Bone regeneration was examined histologically and histomorphometrically. Angiogenesis was assessed by immunohistochemistry against vascular endothelial progenitor cell marker (CD34) and the microvessel density was calculated.

**Results:**

Exposure of diabetic animals to hyperbaric oxygen resulted in superior bone regeneration and increased endothelial cell proliferation, which were revealed histologically and immunohistochemically, respectively. These results were confirmed by histomorphometric analysis which disclosed a higher percentage of new bone surface area and microvessel density in the study group.

**Conclusions:**

Hyperbaric oxygen has a beneficial effect on bone regenerative capacity, qualitatively and quantitively, as well as the ability to stimulate angiogenesis.

## Background

Hyperbaric oxygen therapy (HBOT) is a treatment technique that involves the inhalation of 100 percent oxygen inside a pressurized chamber at a pressure higher than that of sea level or 1 atmosphere absolute (ATA) [1]. During this treatment approach, the elevated pressure causes oxygen to dissolve in plasma and results in elevated arterial and tissue oxygen tensions, permitting the delivery of more oxygen necessary for the healing of hypoxic or damaged tissues [2].

Currently, there is a constant search for innovative bio-stimulatory techniques to enhance the healing of impaired tissues and reduce possible complications [3]. HBOT is an efficient adjunctive treatment method for managing a wide range of pathological conditions owing to its anti-inflammatory and antibacterial properties, as well as its ability to resolve edema [2, 4].

Moreover, HBOT increases vascular density within the damaged tissues, which improves the availability of oxygen for repair [5]. This process occurs through different mechanisms, including the increased synthesis of several angiogenic growth factors as well as the liberation of endothelial progenitor cells (EPCs) into circulation from the bone marrow [6–9]. The beneficial effects of HBOT on bone repair capacity have been demonstrated in numerous studies, where this therapy has been associated with enhanced proliferation and differentiation of osteoblasts with improved biomineralization, increased alkaline phosphatase activity, and accelerated fracture healing [10–13].

However, the majority of studies that examined the impact of HBOT on diabetes mellitus (DM) were focused on soft tissue healing [14–16]. Type I diabetes mellitus (T1DM) is a metabolic disease resulting from immune-mediated destruction of pancreatic beta cells that produce insulin, which leads to hyperglycemia [17]. Several complications of the skeletal system are associated with DM, including reduction of bone mineral density, increased fracture risk, decreased bone turnover rate as well as a compromised angiogenic process [18–20]. Therefore, investigating adjunctive therapies to reduce these adverse effects of DM on bone regeneration is crucial.

Critical-sized defects (CSDs), which can be caused by trauma, infections, or tumor resection surgeries, are defined as bone defects that will not heal spontaneously without medical intervention. In clinical practice, reconstruction of such defects represents a major challenge [21], especially in diabetics for the previously mentioned reasons. Consequently, in an impaired osteogenic condition, HBOT can be part of the treatment regime in association with bone grafts to regenerate these defects [22]. Beta-tricalcium phosphate (β-TCP) is a synthetic bioceramic bone grafting material. For certain maxillofacial grafting procedures, β-TCP is considered a good substitute for autografts and allografts due to its osteoconductive and osteoinductive properties as well as its cell-mediated resorption [23, 24].

To the best of our knowledge, studies available about using HBOT with alloplastic bone grafts for bone defect regeneration in diabetics are limited. Thus, the present study aims to evaluate the effect of this therapy on the healing of CSDs filled with β-TCP in rats with induced T1DM. However, the null hypothesis proposed that HBOT has no significant effect on bone regeneration of mandibular defects in diabetic rats.

## Materials and methods

The current study’s manuscript follows the ARRIVE (Animal Research: Reporting in vivo experiments) guidelines for the animal research reporting [25].

### Animals and ethical statement

The Research Ethics Committee for Animal Care and Use, Faculty of Dentistry, Alexandria University, Egypt (IORG 0008839-0216-01/2021) has approved this study. We followed all the applicable international, national, and/or institutional guidelines for animal care and use. The animals were obtained from the animal house of the Faculty of Dentistry, Alexandria University, Egypt.

Sixteen healthy adult male albino rats (250–280 g), approximately six months of age, were used in this experiment. They were kept in specially designed cages with wire mesh bottoms, having unrestricted access to standard food and water, in the same controlled laboratory settings of temperature (22–25 °C), good ventilation, and regular light/dark cycle (12/12 h). The cages were cleaned twice daily, because of repeated urination in diabetes.

### Study design and randomization

This study is a controlled experimental animal study. The sample size required for statistical testing was estimated based on calculations made in the Biomedical Informatics and Medical Statistics Department, Medical Research Institute, Alexandria University, Egypt. To calculate a representative sample size, 80% study power and an alpha of 0.05 were used.

Each rat was given a number between 1 and 16. Using a random number generator (Prism G. version 5.04, GraphPad Software Inc: San Diego, CA, USA), animals were allocated into two equal groups as follows:

- Group A (n = 8): Control group, diabetic animals with critical-sized defects filled with β-TCP.
- Group B (n = 8): Study group, diabetic animals with critical-sized defects filled with β-TCP and subjected to HBOT.

### Study setting

The present study was conducted at the animal house of the Faculty of Dentistry, Alexandria University, Egypt. HBOT was performed at the Naval and Underwater Medical Institute, Alexandria, Egypt. The histological procedures were carried out in the histological unit of the department of Oral Biology, Faculty of Dentistry, Alexandria University, Egypt.

### Experimental procedures

#### Induction of type I diabetes mellitus

Animals were kept on 12-h fasting before inducing T1DM. A single (50 mg/kg) intraperitoneal injection of streptozotocin (STZ) (Sigma-Aldrich, St. Louis, MO, USA) was used, dissolved in 0.1 M citrate buffer (4.5 pH) immediately prior to the injection. Blood glucose level analysis was performed to confirm DM after 72 h from the administration of STZ, then on a weekly basis for the duration of the experiment, using OneTouch digital glucometer (LifeScan, Milpitas, CA, USA) and obtaining blood samples from the animals tail vein. Diabetes was regarded as fasting blood glucose levels above 250 mg/dL [3].

#### Surgical procedure

The procedure was done under general anesthesia via intramuscular injection of (7 mg/kg) xylazine 2% (XylaJect, Adwia Co, 10th of Ramadan city, Egypt) and (80 mg/kg) ketamine 10% (Ketamine Alfasan, El Yoser Co, Cairo, Egypt) [26]. Using an aseptic technique, a 15 mm linear incision was made along the line of the posterior right mandibular basal bone. Afterward, a full-thickness mucoperiosteal flap was reflected and a round 4 mm-diameter critical-sized osseous defect was prepared, using a sterile trephine bur (Medesy, JDentalCare, Cairo, Egypt) [3, 27]. After irrigation with sterile saline, the osseous defect was filled with β-TCP bone graft (Nano Gate, Nasr city, Cairo, Egypt). Then, the flap was repositioned and stitched with resorbable sutures (Vicryl, Ethicon, Somerville, NJ, USA) [28]. The operative sites were checked daily, and the rats were monitored for any symptoms. Postoperatively and for 3 days, antibiotic and analgesic were given in the form of 30 mg/kg cefazolin sodium (ALPAC-AL Wady Pharmaceutical, Helwan, Egypt) and 5 mg/kg Carprofen (Adwia Co, 10th of Ramadan city, Egypt) [26].

#### Hyperbaric oxygen therapy

Starting 24 h postoperatively, Group B was subjected to HBOT using a mono-place chamber. Animals were placed in custom-made plastic boxes with oxygen administration tubes [22]. They were subjected to 90-min HBO sessions at 2.4 ATA, five consecutive days per week (from Saturday to Wednesday) for 3 weeks [22, 29]. To prevent barotrauma and discomfort, pressurization and depressurization processes took place over 10 min each. The untreated control animals in group A were kept in the same room outside the hyperbaric chamber.

#### Animal euthanasia

Euthanasia was done after 3 weeks of therapy via the injection of an overdose of barbiturates intravenously (Nembutal, pentobarbital sodium, Akorn Pharmaceuticals Co.) [30]. The mandibles were dissected out and cleaned of soft tissues. The experimental segments with the osseous defects were used for histological evaluation, immunohistochemical and histomorphometric analysis.

#### Histological examination

Specimens were labeled and then fixed instantly in 10% neutral-buffered formalin. Washing, decalcification by 8% tri-chloroacetic acid, dehydration with increasing grades of alcohol, clearing with xylene, then infiltration and embedding in paraffin wax blocks were carried out. Serial tissue sections, 5-μm thick, were cut sagittally through the center of the defect. Staining with Hematoxylin & eosin (H&E) and Gomori trichrome was performed for the general examination of bone regeneration and the evaluation of collagen formation, respectively. Sections were examined by a light microscope (OPTIKA microscopes B-290 series, Ponteranica, Italy) by two different investigators for qualitative histological evaluation.

#### Histomorphometric analysis

Morphometric analysis was performed on H&E-stained sections using the ImageJ software (ImageJ 1.53k, NIH, Bethesda, MD, USA) to calculate the percentage of the newly formed bone surface area within the defects. Three sections were obtained from each sample to be used for quantification by two blinded investigators. Then one image was taken from each section under the same magnification power (X40), via a light microscope (OPTIKA Microscopes B-290 series, Ponteranica, Italy) with an attached camera (OPTIKA Microscopes C-B10, Ponteranica, Italy) and Optika Proview software (OPTIKA Microscopes, version 3.7, Ponteranica, Italy). In each digital image taken, the surface area of a selected region of interest (ROI) was measured and recorded as the total surface area. The area that the marrow spaces and other tissue spaces occupied was also measured. Afterward, the surface area occupied solely by bone was obtained by subtracting these two measurements, and its percentage from the total area was calculated. The mean of the measurements from the three images was determined to be used for statistical analysis.

#### Immunohistochemical analysis and microvessel density

For quantitative assessment of intraosseous microvessel density (MVD), immunostaining against the endothelial progenitor cell marker (CD34) was performed using monoclonal anti-CD34 antibodies (Thermo Scientific, Fremont, CA, USA) [31].

Surveying of sections was initially done at (×100) magnification, followed by a randomized selection of five microscopic regions with the densest concentration of vessels (vascular hot spots) per section. In each image, a standardized counting frame was placed over these areas, and the microvessel number was counted under (×400) magnification using the ImageJ software (ImageJ 1.53k, NIH, Bethesda, MD, USA). A single microvessel was considered as any positively immuno-stained endothelial cells or endothelial cell clusters, clearly separated from the connective tissue elements or adjacent microvessels. This was done by two different observers to avoid bias. The mean value of microvessel number per mm^2^ was used to calculate the MVD based on an established protocol by Weidner et al. [32].

#### Statistical analysis

The obtained quantitative data were subjected to statistical analysis using Student’s *t-*test and expressed as mean values and standard deviations (SD) with a statistical significance level set at 5% (*p* < 0.05). IBM SPSS software package version 20.0 (Armonk, NY: IBM Corp.) was utilized to perform the analysis.

## Results

### Clinical observations

All animals tolerated the hyperbaric oxygen sessions and the surgical procedure well. Transient drowsiness and lack of appetite were observed following anesthesia recovery. They maintained hyperglycemia throughout the experimental period. Weight loss, polyphagia, polydipsia, and polyuria were noted.

### Histological results

#### Group A: Control group

**Fig. 1:**
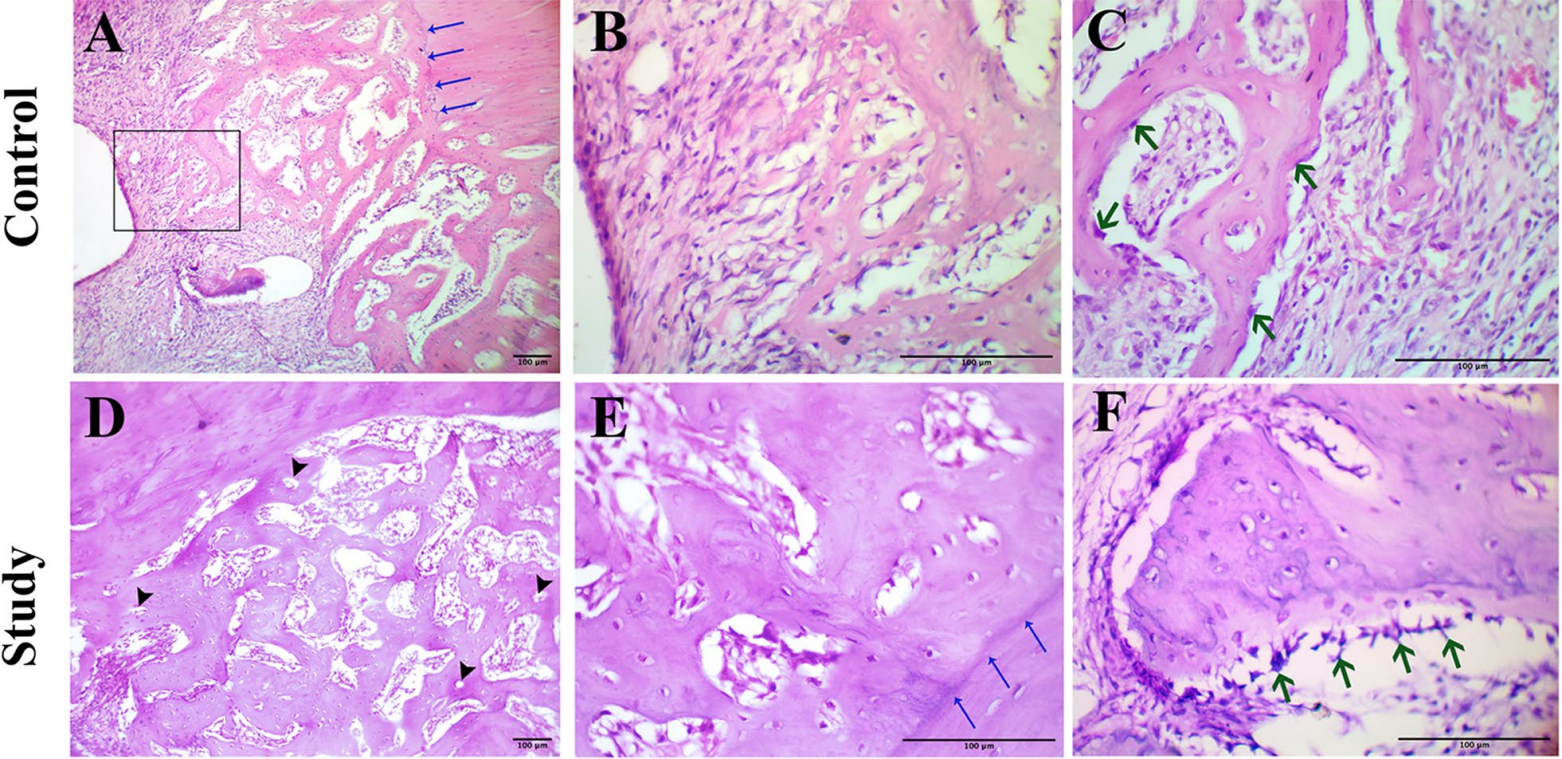
Light micrographs (LM) of H&E stained decalcified sections. **A** and **D** ×100 magnification. **B**, **C**, **E**, **F** ×400 magnification. **A** showing irregular trabeculation of the newly formed bone. The demarcation line between the native and the new bone (blue arrows). **B** higher magnification of the inset in the previous micrograph showing the extension of immature bone formation toward the defect center. **C** showing the discontinuous layer of osteoblasts (green arrows). **D** showing thick and well-organized bone trabeculae with primary osteon formation (arrowheads). **E** showing the interconnectivity and thickness of the trabeculae and their osteointegration with the native bone (blue arrows). **F** showing high activity of osteoblasts (green arrows)

**Fig. 2:**
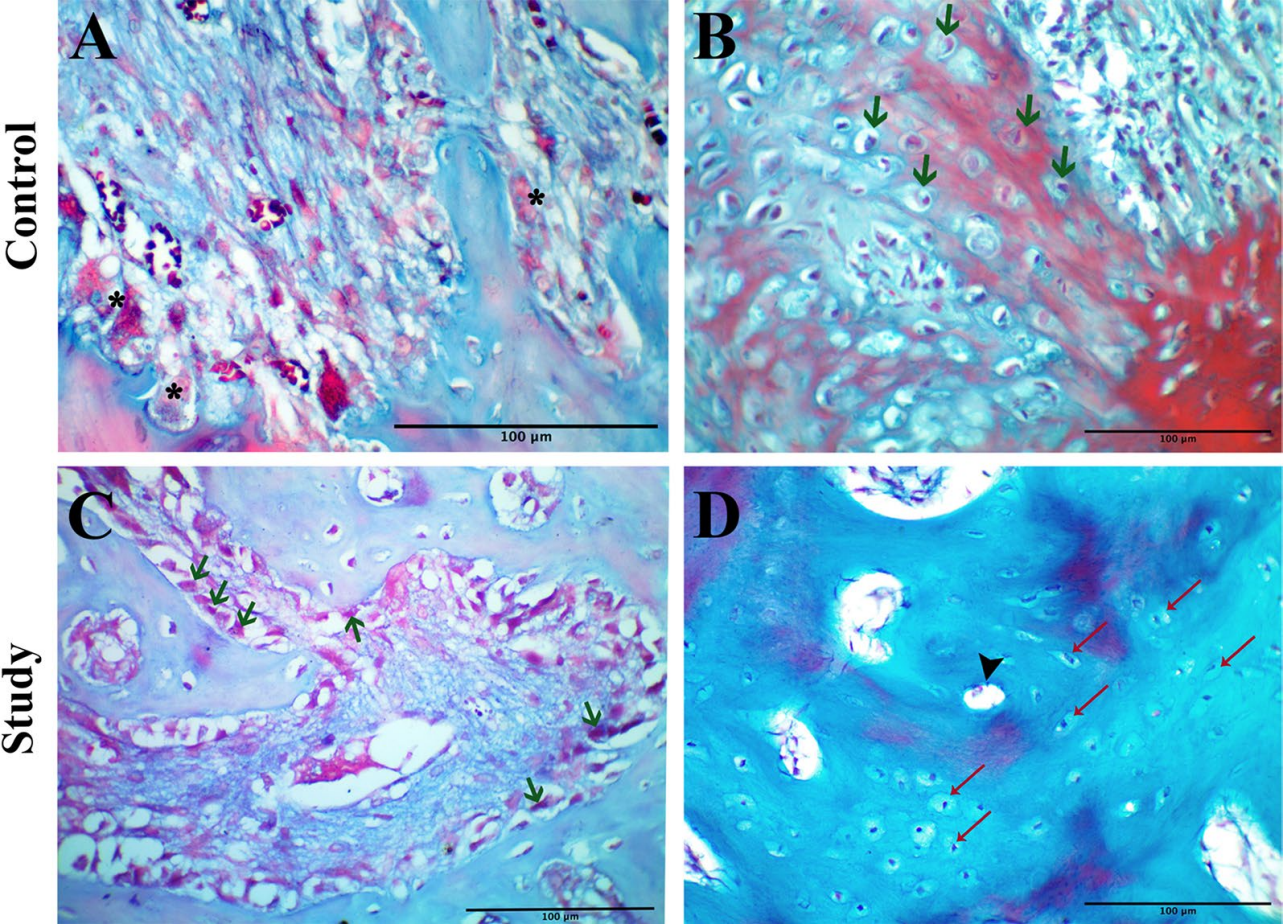
LM of decalcified sections stained with Gomori trichrome stain, ×400 magnification.** A** showing collagen mapping the forming bone trabeculae. Osteoclasts on the surface of the defect margin and the new trabeculae (asterisks).** B** showing bone formation with early trapping of osteoblasts (green arrows). **C** showing an active area of bone formation consisting of collagen and voluminous osteoblasts (green arrows). **D** showing an osteon formation in the regenerated bone (arrowhead) and entrapped osteocytes (red arrows)

Microscopic examination showed the newly formed bone originating from the borders of the defect and extending a short distance toward the defect center in the form of irregular trabeculae in association with fibrous connective tissue of low density and limited vascularity. A discontinuous layer of osteoblasts lined the immature bone trabeculae which contained many large, entrapped osteocytes. A line of demarcation was observed in some areas of the defect boundary between the native and newly formed bone denoting their fusion (Figs. 1, 2).

#### Group B: Study group

A striking picture of active bone formation could be observed occupying most of the defect regions. Together with segments of bone with primary osteon formation, areas of new trabecular bone were seen in the form of intercommunicating trabeculae of better organization than what was observed in group A. The newly formed bone was lined by a continuous layer of voluminous active osteoblasts and had osteocytes that appeared more organized than those of the control group. High vascularity of the bone marrow and the regeneration front was observed. An outstanding osteointegration of the new bone with the native bone was noted in most of the sections examined (Figs. 1, 2).

### Immunohistochemical results

**Fig. 3:**
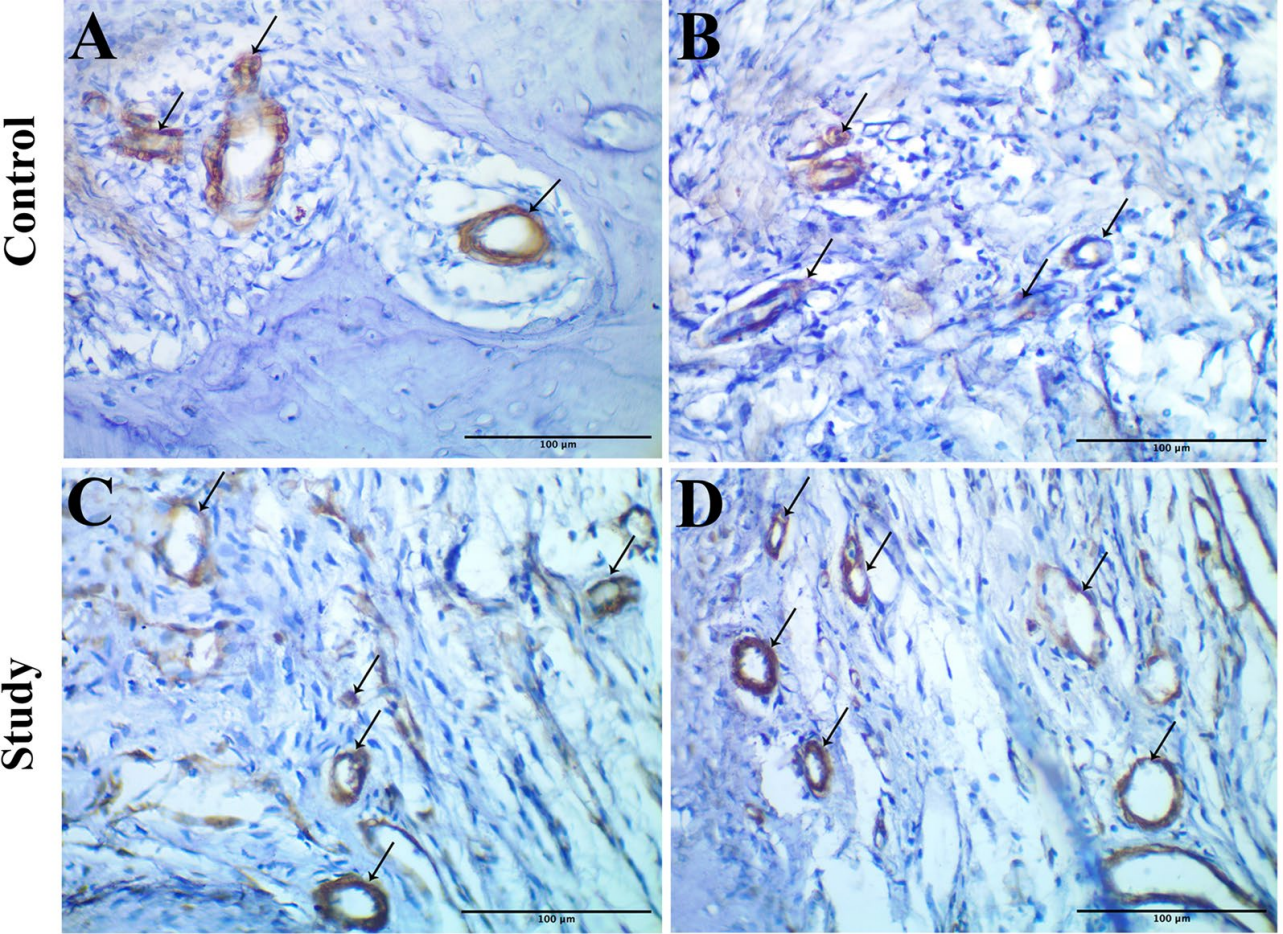
Immunohistochemical micrographs showing the difference in angiogenesis and microvessel content (arrows) between the control group (**A**, **B**) and the study group (**C**, **D**), using CD34 (IHC stain, ×400 magnification)

Endothelial cells acquired yellow–brown color in the current immunostaining procedure, indicating a positive reaction with the antibodies against the endothelial progenitor cell marker (anti-CD34). The Immunohistochemical findings showed that the study group exhibited greater angiogenesis than the control group, observed as an increase in the microvessel content within the regenerating tissues. Any endothelial-lined vessel lumen or endothelial cell cluster appearing yellow–brown was considered to be a single microvessel (Fig. 3).

### Histomorphometric results

#### Newly formed bone surface area

Comparing the study group and the control group, there was a statistically significant difference (*p* < 0.001) regarding the percentage of newly formed bone surface area with mean values of (36.26 ± 4.16%) and (15.55 ± 1.91%), respectively. The percentages of the new bone surface area are demonstrated in Table 1.

**Table 1:** Comparison between the newly formed bone in the control and the study groups

Percentage of new bone surface area	Groups	
	A	B
Min.–Max	13.29–19.05	29.85–43.65
Mean ± SD	15.55 ± 1.91	36.26 ± 4.16
Median	15.15	36.43
t (*p*)	12.799 (< 0.001*)	

t: Student *t*-test, *p*: *p* value for comparing between the studied groups

*Statistically significant at *p* ≤ 0.05

#### Microvessel density (MVD)

The MVD exhibited a higher value in group B (96.87 ± 7.12/mm^2^), treated with HBO, in comparison with group A (73.96 ± 7.12 /mm^2^), with a statistically significant difference between the two groups (*p* = 0.004). The mean values of MVD in the defects are demonstrated in Table 2.

**Table 2:** Comparison between the microvessel density (MVD) of the control and the study groups

Microvessel density	Groups	
	A	B
Min.–Max	66.67–83.33	87.50–104.2
Mean ± SD	73.96 ± 7.12	96.87 ± 7.12
Median	72.92	97.92
t (*p*)	4.554 (0.004*)	

t: Student *t*-test, p: *p* value for comparing between the studied groups

*Statistically significant at *p* ≤ 0.05

## Discussion

In oral and maxillofacial surgery, achieving bone regeneration for defects of critical size is a crucial issue. Although multiple methods have been applied to repair these defects, their incomplete closure or non-union remains a clinical challenge [18].

Hyperbaric oxygen therapy is an effective and relatively safe adjunctive treatment option for the management of a variety of disorders, such as osteoradionecrosis, refractory osteomyelitis, decompression sickness, gas embolism, and intracranial abscess [2]. To the best of our knowledge, the available studies addressing the influence of HBOT on bone regeneration in diabetic models are limited. Diabetic patients are more likely to experience impaired postoperative bone healing, as bone is the primarily affected tissue from hyperglycemia [17]. Therefore, the efficacy of HBOT on bone regeneration in rats with experimentally induced type I diabetes mellitus was assessed in the present study.

Regarding the current research area, CSD is one of the most used in vivo experimental models for the evaluation of bone healing. The posterior mandibular region was selected, in the present study, as an operative site to mimic the maxillofacial defects and the smallest possible CSD of 4 mm diameter was created to reduce the complication risk as much as possible [3, 27].

The current gold standard for grafting is considered the autologous cancellous bone graft transplantation [33]. However, harvesting such grafts can result in morbidity and pain at the donor site and is constrained by availability [34]. Therefore, in some regenerative procedures, bone substitutes or bioceramics became reliable alternative treatment options to overcome these drawbacks [24]. In the present study, β-TCP was chosen as a grafting material to be employed with HBOT for defect regeneration because it is known to be biocompatible, biodegradable, and osteoconductive [23, 24, 28].

The HBOT protocol composed of 90-min sessions at 2.4 ATA once daily for 5 times a week, used in the present study, proved to be effective in enhancing bone regeneration and angiogenesis in the mandibular defects of diabetic animals. These findings are supported by those of Grassmann et al. [22], who used the same HBOT protocol with autogenous bone graft in critical-sized diaphyseal defects and evaluated bone healing radiologically and histologically. They found that the additional HBOT leads to significantly increased bone formation in the defects compared to the treatment with autologous bone graft alone.

In the current study, the histological findings of the study group indicated a highly active bone formation process. The newly formed bone was more mature and had a better organization than what was observed in the control group. This is consistent with the results obtained by Jan et al. [29], who used a similar HBOT regimen for 4 weeks in calvarial critical-sized defects. Their histological analysis revealed significantly more bone in the nongrafted HBO-treated defects than in the nongrafted untreated ones. However, in contrast to our study, the grafted HBO and control defects showed no statistical difference. Moreover, Dias et al. [35] provided histological evidence for accelerated initial bone regeneration in femoral defects of diabetic rats after 7 days of treatment with HBOT, but unlike the current study, no bone graft was applied. This is thought to reveal the importance of using HBO, with or without a grafting material, for the healing of bone defects in a diabetic environment.

It was also observed in the study group that most of the new bone trabeculae were lined with a continuous layer of voluminous and active osteoblasts, an observation thought to confirm that HBOT increases osteoblast formation and activity. These results are supported by the findings of Wu et al. [10] who demonstrated that daily HBO exposure enhances the human osteoblast proliferation and differentiation toward the osteogenic phenotype in vitro*.* In addition, HBOT improved calcium deposition, increased bone nodule formation in terms of number and size, and improved alkaline phosphatase (ALP) activity in the treated cultures. Furthermore, the beneficial effects of HBOT on bone healing have also been reported by Kawada et al. [13] who proved that HBOT stimulates osteoblast proliferation in vivo during fracture healing in mice, as the type 1 collagen alpha 1 (Col1A1) and ALP mRNA levels were considerably higher in the HBO group than those in the control group.

Meanwhile, the control group of the present study showed irregular bone trabeculae with discontinuity of the osteoblast cells, these results are explained by the fact that the exposure of osteoblasts to hyperglycemia reduces their differentiation potential, leading to defective osteoid formation and diminished bone mineral apposition rate [19, 36]. Furthermore, Limirio et al. [37] proved that T1DM reduces collagen maturation and the mineral deposition process, hence decreasing the biomechanical properties of bone, while the exposure to HBOT every 48 h at 2.5 ATA improved the crosslink maturation and increased maximum strength and stiffness in the femurs of diabetic rats. This supports the current findings that HBOT can reduce the deleterious effects of diabetes on bone repair.

The histomorphometric analysis in the current experiment confirmed the histological observations, revealing a statistically significant difference in the percentages of the regenerated bone surface area between the study and the control groups. This agrees with the results of Park et al. [38] who created an irradiated calvarial bone defect model, where their histomorphometric analysis showed significantly higher bone formation in the groups treated with HBO compared to the controls.

Due to the tight connection between bone regeneration and neovascularization, an examination of the effect of HBOT on angiogenesis was also studied in the present experiment. It is commonly acknowledged that endothelial damage and reduced endothelium-dependent vasodilation are associated with hyperglycemia [39]. Moreover, several studies proved that diabetes mellitus can negatively impact the performance and activity of different adult stem cell lineages as well as vascular progenitor cells [40–42].

In the present study, the immunostaining with anti-CD34 disclosed a considerably higher number of positive cells in the study group. This finding is supported by the previous results of Pedersen et al. [12], who proved that HBOT stimulates vascularization in a calvarial defect model using endothelial cell surface marker CD31.

The current morphometric analysis concerning the MVD revealed a higher value in group B compared to group A, with a statistically significant difference. This result is thought to prove the significant influence of HBOT on angiogenesis, which is consistent with the findings presented by Yeh et al. [43]. They provided histological confirmation for improved neovascularization at the bone-tendon interface in rabbits treated with HBO, where they assessed and counted the number of blood vessels microscopically in six areas of a bone-tendon junction.

The exact mechanisms by which HBO achieves its beneficial angiogenic action are not completely investigated. However, it was found that HBOT stimulates the release of EPCs and increases the production of angiogenic factors such as vascular endothelial growth factor (VEGF) [7, 9, 44]. furthermore, a genome-wide microarray analysis was conducted by Godman et al. [45] on human microvascular endothelial cells subjected to HBOT. Their findings confirmed HBO’s potent cytoprotective and antioxidant effects on endothelial cells as well as the activation of growth-promoting genes.

Finally, all the previous findings revealed that the study group’s bone regeneration and angiogenesis were significantly greater than those of the control group. These results have rejected the null hypothesis proposed before conducting the experiment. However, this study has some limitations because the impact of HBOT on the regeneration of mandibular critical-sized defects was investigated for a short interval, further studies are demanded to evaluate its long-term effect.

## Conclusions

In conclusion, the current study verifies that the application of HBOT combined with β-TCP bone graft can effectively enhance bone regeneration and increase the proliferation of endothelial cells and intraosseous microvessel density in bone defects of diabetic animals. Therefore, an improved clinical outcome is expected with HBOT in the quest to restore large osseous defects in an impaired osteogenic condition such as diabetes mellitus.

## Data Availability

All datasets and materials used and/or analyzed during the current study are included in this published article.

## Abbreviations

HBOT: Hyperbaric oxygen therapy
ATA: Atmosphere absolute
EPCs: Endothelial progenitor cells
DM: Diabetes mellitus
T1DM: Type I diabetes mellitus
CSDs: Critical-sized defects
β-TCP: Beta-tricalcium phosphate
STZ: Streptozotocin
H&E: Hematoxylin and eosin
ROI: Region of interest
MVD: Microvessel density
ALP: Alkaline phosphatase
Col1A1: Type 1 collagen alpha 1
VEGF: Vascular endothelial growth factor
